# *Astragalus baimaensis* (Fabaceae), a new species from Yunnan, China

**DOI:** 10.3897/phytokeys.278.191151

**Published:** 2026-07-30

**Authors:** Qi-Yin Chen, Hai-Tao Ma, Jie-Hua Rao, Hai-Feng Li, Bei Jiang, Zhuo Zhou, Yong-Zeng Zhang

**Affiliations:** 1 Yunnan Key Laboratory of Screening and Research on Anti-pathogenic Plant Resources from Western Yunnan, Dali 671000, China Yunnan Key Laboratory for Plant Diversity and Biogeography, Kunming Institute of Botany, Chinese Academy of Sciences Kunming China; 2 College of Pharmacy, Dali University, Dali 671000, China College of Pharmacy, Dali University Dali China; 3 Yunnan Key Laboratory for Plant Diversity and Biogeography, Kunming Institute of Botany, Chinese Academy of Sciences, Kunming 650201, Yunnan, China Yunnan Key Laboratory of Screening and Research on Anti-pathogenic Plant Resources from Western Yunnan Dali China

**Keywords:** *Astragalus baimaensis*, morphology, new species, phylogenetic analysis

## Abstract

Based on an integrative analysis of morphological traits and molecular phylogenetic evidence, a new species of *Astragalus* (Fabaceae), *A. baimaensis* B. Jiang & Y. Z. Zhang, discovered during field investigations in the Baima Mountain region, Deqin County, Yunnan Province, China, is formally described. This species is defined by a unique combination of diagnostic morphological characters that unequivocally distinguish it from all previously documented congeners: it is completely glabrous throughout, with narrowly oblong leaflets, yellow flowers, and obliquely ellipsoid, glabrous, inflated legumes. The full chloroplast genome of *A. baimaensis* is 124,633 bp in length. Phylogenetic reconstructions based on plastid genomic data resolve *A. baimaensis* as sister to *A. tongolensis*, whereas analyses of the combined nuclear ITS and plastid (*rbcL*, *matK*) multilocus dataset support a closer evolutionary affinity between *A. baimaensis* and *A. chilienshanensis*. Despite these close phylogenetic relationships, the new species exhibits a distinct set of consistent morphological differences that clearly distinguish it from both putative relatives. The results highlight the considerable taxonomic value of variation in ovary pubescence within *Astragalus* and confirm that the presence or absence of trichomes on the ovary represents a stable diagnostic trait for species delimitation and infrageneric classification in this genus. This study adds to the known diversity of *Astragalus* and provides data for future phylogenetic and ecological studies.

## Introduction

*Astragalus* L. (Fabaceae) is the most species-rich genus of seed plants. It comprises approximately 3,000 species worldwide, reflecting remarkable taxonomic diversity (Folk et al. 2024). In addition to their unparalleled species diversity, members of this genus play a fundamental ecological role by driving biological nitrogen fixation and facilitating soil amelioration (Wang et al. 2015). A large proportion of *Astragalus* species also have well-documented medicinal relevance, as they synthesize a suite of characteristic bioactive compounds, including polysaccharides, saponins, and flavonoids, that underpin a broad spectrum of validated pharmacological activities (Fu et al. 2014; Durazzo et al. 2021). China harbors a documented total of 401 *Astragalus* species, 221 of which are nationally endemic (Xu and Podlech 2010). Although the genus occupies a wide geographic range across China, its distribution is spatially heterogeneous, defining two clearly delimited centers of species diversity. The first and, by far, the most taxonomically rich hotspot extends across the Hengduan Mountains and the Qinghai–Tibet Plateau, centered in the interconnected high-mountain and deep-gorge landscapes of northwestern Yunnan, western Sichuan, eastern Xizang, and Qinghai (Wen et al. 2014). This region contains an extraordinary concentration of endemic lineages and morphologically distinct infrageneric groups, where extreme elevational gradients spanning dry, hot valley bottoms to exposed alpine scree habitats have acted as a powerful driver of rapid ecological speciation (Zhang et al. 2009). The second major distribution center is situated in the arid biomes of northwestern China, covering Xinjiang, Inner Mongolia, Gansu, and Ningxia, where *Astragalus* species are predominantly adapted to steppe, desert, and Gobi habitats, forming structurally unique xerophytic plant assemblages (Sui et al. 2024).

Although more than 3,000 species of *Astragalus* have been identified, the genus-wide infrageneric classification system remains widely debated and far from fully resolved (Osaloo et al. 2003). This taxonomic challenge can be primarily attributed to three interacting intrinsic and extrinsic drivers: extraordinarily intricate patterns of continuous morphological variation across lineages, rampant interspecific hybridization events that blur species boundaries, and the persistent undersampling of vast, remote mountain and arid regions across the genus’s extensive intercontinental distribution range (Scherson et al. 2008; Xie et al. 2016). The combined effects of these factors have greatly hindered the construction of a robust, stable taxonomic framework, leaving substantial gaps in the comprehensive documentation of *Astragalus* biodiversity. In recent years, transformative advances in integrative taxonomic methodologies—especially the widespread adoption of molecular phylogenetic inference and high-resolution micromorphological characterization—have substantially accelerated the discovery and formal delimitation of numerous previously overlooked cryptic and geographically restricted endemic species (Padial et al. 2010). Well-documented recent examples include *A. remotispicatus* Bagheri & Maassoumi (Bagheri et al. 2016), *A. oksutdagensis* H. Duman & Karaman (Karaman Erkul et al. 2022), and *A. kuzehrashensis* Karbalaei, Bagheri & Maassoumi (Karbalaei et al. 2025). These targeted taxonomic revisions and new species discoveries have continuously refined the understanding of species richness and evolutionary diversification trajectories within this hyperdiverse genus.

During a systematic botanical field survey conducted in July 2024 in the alpine zone of Baima Mountain, Deqin County, Shangri-La, Yunnan Province, China (28°20'10.896"N, 99°4'58.332"E), a morphologically distinctive perennial herb assignable to the genus *Astragalus* was encountered. Subsequent integrative taxonomic investigations confirmed this plant to be an undescribed new species, formally named here *A. baimaensis*. The new taxon is immediately distinguishable from all congeneric species by its fully glabrous phenotype: no trichomes are present on the leaves, bracts, calyces, petals, or ovary, and all surfaces are uniformly smooth. Additionally, its legume exhibits a suite of unique morphological synapomorphies that clearly distinguish it from all currently known *Astragalus* species. Through meticulous specimen dissection, comprehensive micromorphological comparisons, and robust phylogenetic analyses—using both concatenated gene fragments (ITS, *rbcL*, and *matK*) and the complete chloroplast genome—the taxon was identified and confirmed as a novel species within *Astragalus*.

This work presents a fully validated taxonomic description integrating morphological diagnostics and phylogenomic evidence for the novel taxon *Astragalus baimaensis*, explicitly resolving its phylogenetic affiliation within the genus. The new taxon bridges a long-recognized phylogenetic gap in the undersampled alpine biodiversity hotspot of the Hengduan Mountains, refining the resolution of species diversity in the eastern Himalaya–Hengduan Mountains regional flora. These outputs not only deepen the systematic understanding of *Astragalus* diversification but also provide critical baseline data to underpin future phylogenetic investigations, targeted conservation interventions, and sustainable utilization frameworks for *Astragalus* plant resources.

## Materials and methods

### Plant sampling and morphological observation

**Figure 1.**
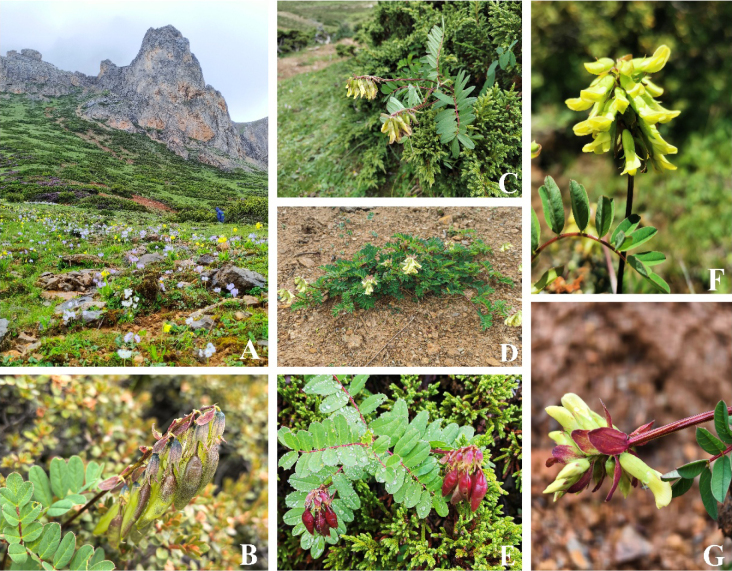
Habitat and morphology of *Astragalus baimaensis*. **A**. Habitat; **B**. Immature pods; **C, D**. Plants in their habitat; **E**. Mature pods; **F**. Flower, front view; **G**. Flower, lateral view.

**Figure 2.**
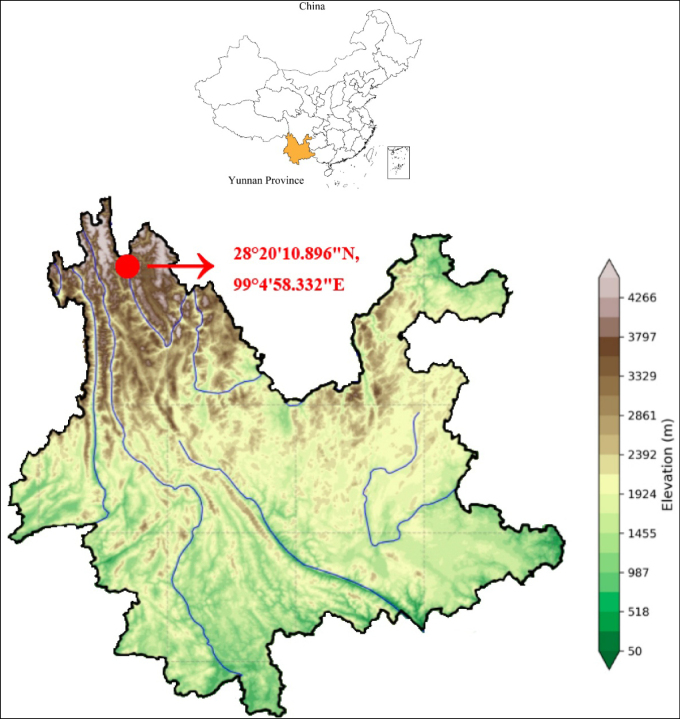
Distribution locality of *Astragalus baimaensis*, indicated by a red dot on the topographic map of Yunnan Province.

In July 2024 and August 2025, specimens and living material of the newly discovered species, as well as those of *Astragalus tongolensis*, were collected in the Baima Mountain region of Deqin County, Shangri-La, Yunnan Province, China (coordinates: 28°20'10.896"N, 99°4'58.332"E). The morphological characteristics and corresponding measurement data of both the new species and its closely related taxa were meticulously recorded based on observations of more than 50 living plants and preserved specimens (Figs 1, 2). The type specimens of this new species are currently deposited in the Herbarium of the Kunming Institute of Botany, Chinese Academy of Sciences (KUN). The accession number of the holotype is KUN1654853, whereas that of the isotype is KUN1654854 (Suppl. material 1: figs S1, S2). The paratype specimens are assigned the accession numbers KUN1654855 and KUN1654856 (Suppl. material 1: figs S3, S4).

### DNA extraction and sequencing

Genomic DNA from *Astragalus baimaensis* and *A. tongolensis* was extracted from silica-gel-dried leaf tissue using a modified cetyltrimethylammonium bromide (CTAB) method (Doyle 1987). The quality and concentration of the DNA were assessed using 1.0% agarose gel electrophoresis and spectrophotometry (Bio-Rad, Hercules, USA). Subsequently, the genomic DNA was fragmented into segments of approximately 350 bp to facilitate library construction. Sequencing was performed on the DNBSEQ-T7 platform, ensuring high-throughput and reliable data generation. Following sequencing, low-quality reads were filtered using fastp v.0.23.2 (Chen 2023). All sequencing procedures in this study were performed by Wuhan Bena Biotechnology Co. Ltd.

The amplification reaction system for the ITS sequence was prepared as follows: 2× Taq PCR Master Mix (10 μL), two ITS primers (0.3 μL), template DNA (1 μL), and ddH_2_O (8.4 μL). PCR amplification was conducted under the following conditions: initial denaturation at 95 °C for 2 min; 35 cycles of denaturation at 94 °C for 40 s, annealing at 55 °C for 50 s, and extension at 72 °C for 55 s; and a final extension at 72 °C for 8 min. The primer sequences were as follows: ITS5: 5’-GGA AGG AGA AGT CGT AAC AAG G-3’; ITS4: 5’-CTT TTC CTC CGC TTA TTG ATA TG-3’. The entire ITS region was sequenced by Sangon Biotech (Shanghai) Co., Ltd.

### Assembly and annotation of plastid genomes and ITS sequence

The high-quality clean reads were assembled *de novo* using GetOrganelle v.1.7.7.1 with default parameters (Jin et al. 2020), ultimately yielding a circular chloroplast genome. The chloroplast genome was annotated using the online tool CPGAVAS2 (Shi et al. 2019). Additionally, two chloroplast DNA sequences (*rbcL* and *matK*) used for phylogenetic analysis were extracted. Genome maps were visualized using OGDRAW (Greiner et al. 2019). For tRNA annotation of the chloroplast genome, tRNAscan-SE was employed (Lowe and Eddy 1997), whereas rRNA annotation was performed using the BLASTN program (Chen et al. 2015). The annotation results were manually corrected using the CPGView online platform (Liu et al. 2023) and the Apollo genome editor (Lewis et al. 2002). The original ITS DNA sequences were assembled using the SeqMan program (DNASTAR, Lasergene) (Clewley 1995). Finally, the ITS sequences of *Astragalus baimaensis* and *A. tongolensis*, along with the fully annotated chloroplast genome sequences, were submitted to GenBank under the accession numbers PX747761.1, PX747760.1, PX754581.1, and PX754582.1.

### Phylogenetic analysis

To robustly assess the phylogenetic affiliation of the new species *Astragalus baimaensis*, two complementary approaches to phylogenetic inference were implemented: a multilocus concatenation analysis using the standard plant barcode set (ITS, *rbcL*, and *matK*) and a phylogenomic analysis based on whole chloroplast genome sequences. All sequence accessions were obtained from the NCBI public repository. The three-locus matrix retained 40 ingroup samples (Suppl. material 1: table SS1), whereas the plastome dataset comprised 45 accessions (Suppl. material 1: table SS2), with *Caragana arborescens* Lam. and *C. jubata* (Pall.) Poir. used as consistent outgroup taxa in both analytical pipelines (Suppl. material 1: tables S1, S2). The sampling limitations of this study are explicitly acknowledged: the overall pool of publicly deposited complete *Astragalus* chloroplast genomes in NCBI is restricted to approximately 50 species, and full-length sequences for the three barcodes also lack sufficient taxonomic coverage, inevitably constraining the taxon richness of the two final datasets.

All sequences were aligned using MAFFT v.7.313 (Katoh and Standley 2013), and the aligned ends were trimmed using trimAl v.1.4 with the automated1 parameter (Capella-Gutiérrez et al. 2009). Subsequent analyses were conducted in PhyloSuite v.1.2.3 (Zhang et al. 2019). The ITS, *rbcL*, and *matK* sequences were concatenated into a single combined dataset for further phylogenetic analysis. The best-fit nucleotide substitution models for each partition (ITS, *rbcL*, and *matK*) were independently evaluated under a partitioned scheme for both maximum likelihood (ML) and Bayesian inference (BI) analyses. The selected models were as follows: ITS—K2P+F+G4 (ML)/SYM+G (BI); *rbcL*—K3P+F+I (ML)/GTR+I+G (BI); and *matK*—TPM3+F (ML)/GTR+G (BI). Additionally, for the complete chloroplast genome alignment, the optimal substitution models were identified as GTR+G4+F (ML) and GTR+I+G+F (BI) using ModelFinder, which is implemented in PhyloSuite.

Phylogenetic reconstruction was performed using both ML and BI methods. For the ML analysis, IQ-TREE v.2.2.0 was employed (Nguyen et al. 2014), and node support was assessed using 1,000 bootstrap replicates (Minh et al. 2013). In parallel, the BI analysis was conducted using MrBayes v.3.2.7 (Ronquist et al. 2012). Two independent Markov chain Monte Carlo simulations were run for 1,000,000 generations, with the initial 25% of samples discarded as burn-in to ensure convergence. The final phylogenetic trees were visualized, optimized, and edited using FigTree v.1.4.4 (Rambaut 2018).

## Results

### Chloroplast genome structure and characteristics of the novel species

**Figure 3.**
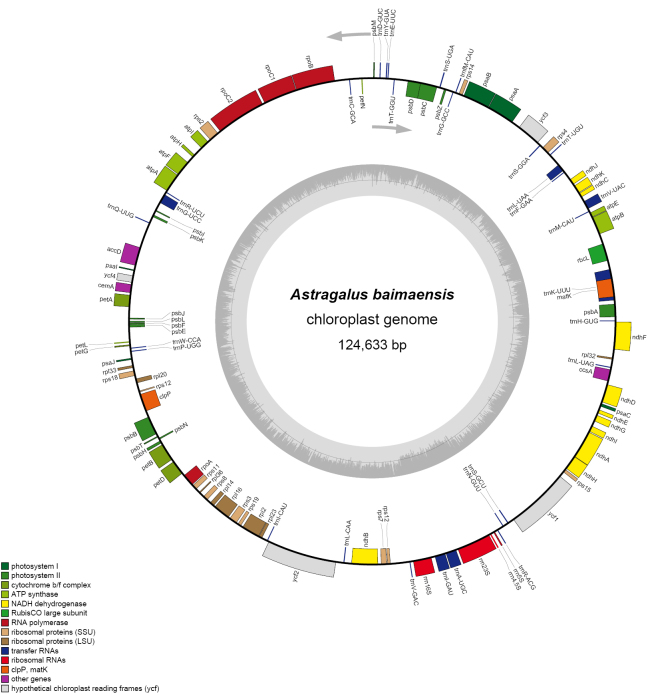
Chloroplast genome map of *Astragalus baimaensis*. Genes within the circle are transcribed clockwise, whereas genes outside the circle are transcribed counterclockwise. Different functional gene groups are represented by different colors. The GC content is displayed in the dotted area within the inner circle.

The chloroplast genome of *Astragalus baimaensis* is a single circular DNA molecule with a total length of 124,633 bp and a GC content of 34.01% (Fig. 3). This genome exhibits a unique structure characterized by a large single-copy region directly connected to a small single-copy region, without the typical inverted repeat regions. Genome annotation revealed a total of 110 genes, including 76 unique protein-coding genes, 30 tRNA genes, and four rRNA genes. Functionally, these genes can be classified into four distinct categories: photosynthesis-related genes, self-replication-related genes, genes associated with other functions, and genes with currently unknown roles (Table 1).

**Table 1.** Annotation information for the chloroplast genome of *Astragalus baimaensis*.

**Category**	**Gene group**	**Gene name**
Photosynthesis	Subunits of photosystem I	*psaA, psaB, psaC, psaI, psaJ*
	Subunits of photosystem II	*psbA, psbB, psbC, psbD, psbE, psbF, psbH, psbI, psbJ, psbK, psbL, psbM, psbN, psbT, psbZ*
	Subunits of NADH dehydrogenase	ndhA*, ndhB*, ndhC, ndhD, ndhE, ndhF, ndhG, ndhH, ndhI, ndhJ, ndhK
	Subunits of cytochrome b/f complex	petA, petB*, petD*, petG, petL, petN
	Subunits of ATP synthase	atpA, atpB, atpE, atpF*, atpH, atpI
	Large subunit of rubisco	*rbcL*
Self-replication	Proteins of large ribosomal subunit	rpl14, rpl16*, rpl2*, rpl20, rpl23, rpl32, rpl33, rpl36
	Proteins of small ribosomal subunit	rps11, rps12*, rps14, rps15, rps18, rps19, rps2, rps3, rps4, rps7, rps8
	Subunits of RNA polymerase	rpoA, rpoB, rpoC1*, rpoC2
	Ribosomal RNAs	*rrn16S, rrn23S, rrn45S, rrn5S*
	Transfer RNAs	trnA-UGC*, trnC-GCA, trnD-GUC, trnE-UUC, trnF-GAA, trnG-GCC, trnG-UCC*, trnH-GUG, trnI-CAU, trnI-GAU*, trnK-UUU*, trnL-CAA, trnL-UAA*, trnL-UAG, trnM-CAU, trnN-GUU, trnP-UGG, trnQ-UUG, trnR-ACG, trnR-UCU, trnS-GCU, trnS-GGA, trnS-UGA, trnT-GGU, trnT-UGU, trnV-GAC, trnV-UAC*, trnW-CCA, trnY-GUA, trnfM-CAU
Other genes	Maturase	*matK*
	Protease	clpP**
	Envelope membrane protein	*cemA*
	Acetyl-CoA carboxylase	*accD*
	*c*-type cytochrome synthesis gene	*ccsA*
Genes of unknown function	Conserved hypothetical chloroplast ORF	ycf1, ycf2, ycf3**, ycf4

Note: * indicates intron-containing genes; Gene*: gene with one intron; Gene**: gene with two introns.

### Phylogenetic analysis

In this study, the phylogenetic relationships of the new species were reconstructed using both complete chloroplast genome data and combined sequence fragment data. Both analytical approaches provided strong support for the inclusion of the new species within the genus *Astragalus*.

**Figure 4.**
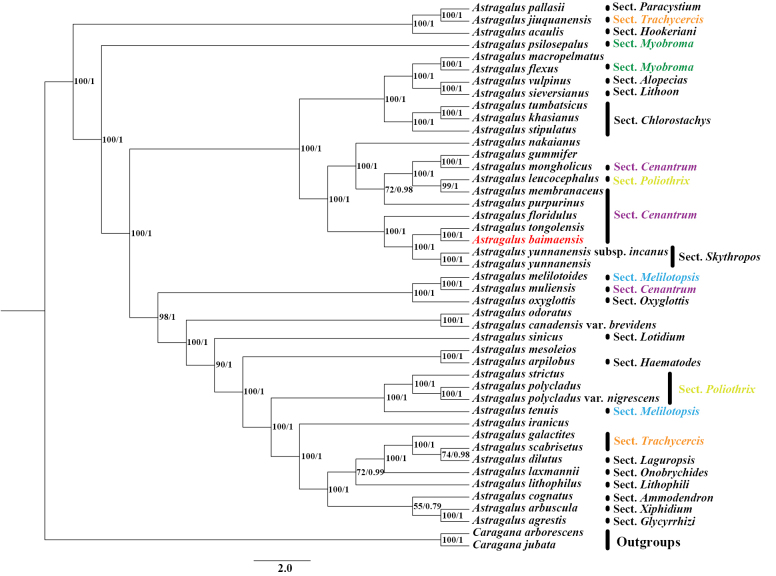
ML and BI phylogenetic tree of 45 Fabaceae species based on complete chloroplast genomes. Node values indicate MLBS (left) and BIPP (right). *Astragalus baimaensis* is highlighted in red. Sectional labels in colors other than black and red indicate nonmonophyletic groups that are discordant with the sectional treatments in Flora of China; all remaining taxa are shown in black.

In the phylogenetic analysis based on chloroplast genome data from 45 accessions, the tree topologies generated by ML and BI were highly congruent. Consequently, the ML tree was selected to represent the phylogenetic results, with branch support indicated by ML bootstrap values (BS) and BI posterior probabilities (PP). In the phylogenetic tree, the sampled *Astragalus* species were taxonomically assigned to 20 sections as circumscribed in Flora of China. However, the vast majority of these traditionally delimited sections were not recovered as reciprocally monophyletic assemblages in the phylogenetic tree: species attributed to the same infrageneric section frequently did not form a single, statistically supported monophyletic lineage. For example, sect. Cenantrum was partitioned into three deeply divergent, phylogenetically distinct, non-sister clades, whereas sect. Poliothrix was similarly separated into two independent lineages (Fig. 4). Only a very small subset of infrageneric sections defined in the Flora of China classification system demonstrated full monophyletic congruence with the molecular phylogenetic inferences. Two of these unambiguous monophyletic units were sect. Chlorostachys, represented here by *Astragalus khasianus*, *A. stipulatus*, and *A. tumbatsicus*, and sect. *Skythropos*, represented by *A. yunnanensis* and A. yunnanensis subsp. incanus (Fig. 4). Most critically, the phylogenomic analyses robustly placed the newly described *A. baimaensis* in a fully supported sister position to *A. tongolensis* within one of the three segregated clades of the conventionally defined sect. Cenantrum (BS/PP = 100/1.00; Fig. 4).

**Figure 5.**
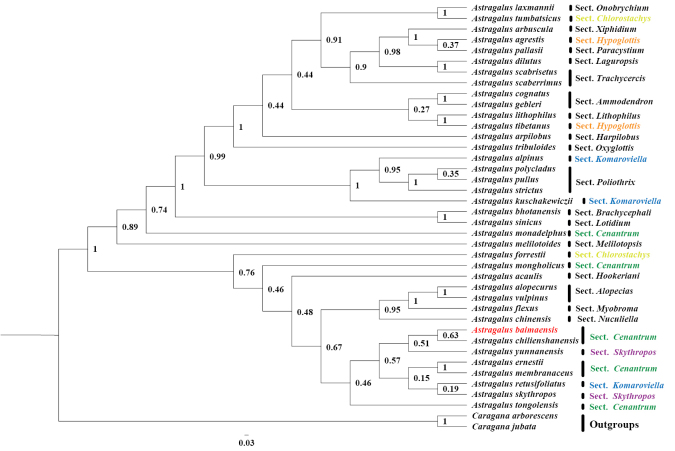
BI phylogenetic tree of 40 Fabaceae species based on concatenated ITS, *rbcL*, and *matK* sequences. Node values indicate BIPP. *Astragalus baimaensis* is highlighted in red. Sectional labels in colors other than black and red indicate nonmonophyletic groups that are discordant with the sectional treatments in Flora of China; all remaining taxa are shown in black.

**Figure 6.**
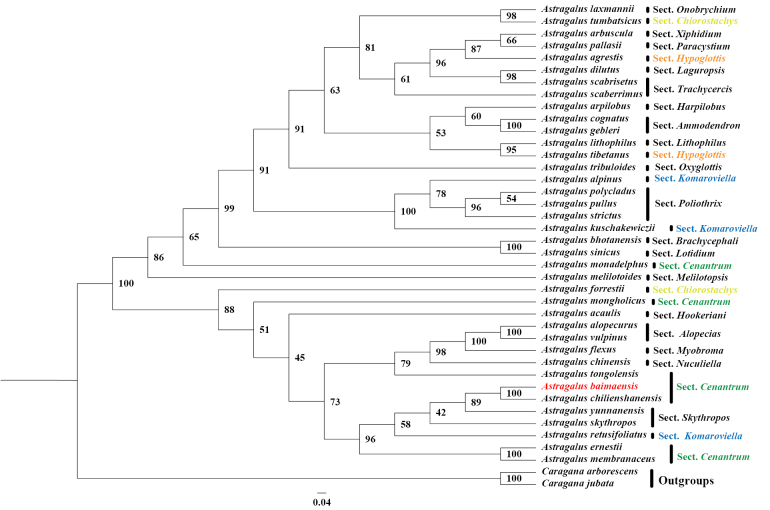
ML phylogenetic tree of 40 Fabaceae species based on combined ITS, *rbcL*, and *matK* sequences. Node values indicate MLBS. *Astragalus baimaensis* is highlighted in red. Sectional labels in colors other than black and red indicate nonmonophyletic groups that are discordant with the sectional treatments in Flora of China; all remaining taxa are shown in black.

Phylogenetic analyses were conducted using a combined dataset of fragment sequences, including ITS, *rbcL*, and *matK*, encompassing a total of 40 *Astragalus* species. Although minor topological incongruence was observed between the ML and BI trees, both analytical frameworks unambiguously resolved *A. baimaensis* as the immediate sister lineage to *A. chilienshanensis*, a species taxonomically placed within sect. Cenantrum (Figs 5, 6). Notably, the BI phylogeny recovered this sister-species relationship with relatively low nodal support (PP = 0.63; Fig. 5), whereas the ML analysis yielded maximum statistical support for the identical phylogenetic placement (BS = 100; Fig. 6).

### Taxonomic treatment

#### Astragalus baimaensis

Taxon classificationPlantaeFabalesFabaceae

B. Jiang & Y.Z. Zhang sp. nov.

E333FF42-80CB-5C91-B06C-00490C2BFAB0

urn:lsid:ipni.org:names:77393449-1

##### Type.

China • Yunnan Province, Shangri-La, Deqin County, Baima Mountain, hillside; 28°20'10.896"N, 99°4'58.332"E; flowering and fruiting, alt. 4,200–4,400 m; 17 July 2024; *Y. Z. Zhang, D. Lei, Q. Y. Chen, H. T. Ma 20240717-1* [holotype KUN (barcode KUN1654853, Suppl. material 1: fig. S1); Isotype KUN (barcode KUN1654854, Suppl. material 1: fig. S2)].

##### Paratype.

The same locality, flowering and fruiting, 7 Aug. 2025, B. Jiang. KUN1654855, KUN1654856 (Suppl. material 1: figs S3, S4).

##### Diagnosis.

**Figure 7.**
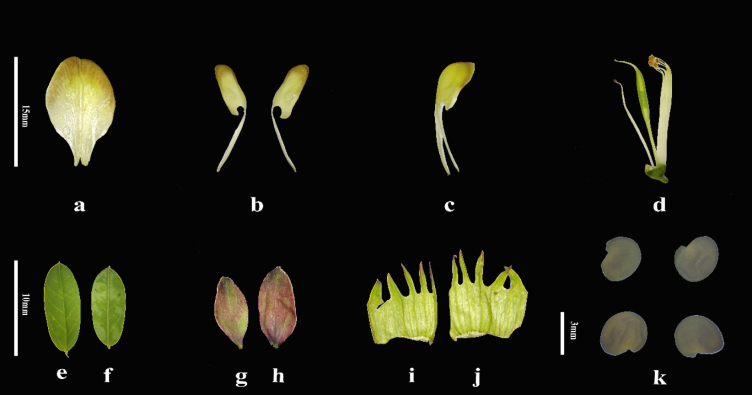
Anatomical illustration of *Astragalus baimaensis*. **a**. Standard; **b**. Wings; **c**. Keel; **d**. Ovary and stamens; **e**. Adaxial surface of the leaflet; **f**. Abaxial surface of the leaflet; **g**. Adaxial surface of the bract; **h**. Abaxial surface of the bract; **i**. Inner surface of the calyx; **j**. Outer surface of the calyx; **k**. Seeds.

The new species stems several, glabrous; leaflets mostly obovate, narrowly oblong, glabrous on both surfaces, apex obtuse and mucronulate; bracts, bracteoles and calyx glabrous on both surfaces; petals green-yellow to yellow, wings and keel ca. 14 mm, standard ca. 15 mm, ovary glabrous; legumes, pendulous, obliquely ellipsoid, 20–30 mm × 6–10 mm, inflated, glabrous, with a stipe 10–14 mm at base are clearly distinguishable to other *Astragalus* species (Figs 1, 7).

##### Description.

***Plants*** 20–50 cm tall. ***Stems*** mostly several, erect, glabrous, angulate, 2–6 mm in diameter. ***Leaves*** 3–9 cm long; stipules triangular or ovoid, green to red purple, ca. 10–15 × 4–8 mm, glabrous; petiole 5–10 mm long, glabrous; leaflets 11–17, obovate, narrowly oblong, 10–22 × 4–8 mm, glabrous on both surfaces, base asymmetric, apex obtuse and mucronulate. ***Racemes*** 8- to many-flowered, elongate after anthesis; peduncle 5–15 cm long, red purple, sulcate, glabrous; bracts oblong or ovoid, green to red purple, 8–15 mm long, glabrous; bracteoles adnate to the calyx oblong or obovate, glabrous, green to red purple, ca. 5–10 × 2–4 mm; pedicels glabrous ca. 2.5 mm. ***Calyx*** campanulate, ca. 7 mm long, glabrous, green to red purple and turn red purple at fruiting; unequal lobes subulate, ca. 3 mm long, glabrous. ***Petals*** green-yellow to yellow, standard ca. 15 × 8 mm, obovate, without distinct claw, apex emarginate; wings ca. 14 mm long, claw ca. 8 mm long, auricle ca. 1 mm long, blade oblong, ca. 6 × 2.5 mm, apex rounded; keel ca. 14 mm long, claw ca. 8 mm long. ***Stamen*** diadelphous filaments glabrous, anthers round. ***Ovary*** with a stipe ca. 1.5 mm, glabrous; style and stigma glabrous. ***Legumes*** inflated, obliquely ellipsoid, with a stipe 10–14 mm at base, 20–30 × 6–10 mm, with a beak 2 mm; valves membranous, glabrous, becoming red-purple with age and red-purple at maturity.

##### Phenology.

Flowering and fruiting from Jun. to Sept.

##### Distribution and habitat.

*Astragalus baimaensis* is endemic to the alpine zone of Baima Mountain, Deqin County, southwestern Yunnan, southwestern China, growing among alpine shrubs or in alpine meadows at elevations of 4,200–4,400 m (Figs 1, 2).

##### Taxonomic notes.

The newly described *Astragalus baimaensis* exhibits close morphological similarity to the sympatric *A. tongolensis* but can be immediately distinguished by its leathery or papery leaflets that are glabrous on both surfaces, accompanied by a fully glabrous calyx, ovary, and legume. Another completely glabrous congener, *A. luculentus*, can be easily distinguished from the new species by a combination of independent characters: *A. luculentus* has larger leaves that are 25–30 cm long with 8–11 cm long petioles, a standard ca. 25 mm long, and subsessile, straight, incompletely bilocular legumes with thick, leathery valves.

##### Conservation status.

*Astragalus baimaensis* is currently known exclusively from Baima Mountain, with an estimated area of occupancy of less than 500 km^2^, which meets the requirements for the Endangered (EN) category under IUCN Red List criterion B2. This taxon occurs at fewer than five threat-defined locations (criterion B2a) and faces a continuing decline in habitat quality driven by anthropogenic disturbances and intensified climate warming—two stressors with disproportionately severe effects on high-altitude alpine species (criterion B2b(iii)). Classification of this new species as Endangered (EN) under the IUCN Red List Categories and Criteria (2012) is therefore recommended.

##### Etymology.

The specific epithet refers to the type locality, Baima Mountain, Yunnan Province, China.

## Discussion

### Chloroplast genome structure of *Astragalus baimaensis*

In contrast to most angiosperms, the newly reported *Astragalus baimaensis* has undergone complete loss of the inverted repeat (IR) regions, a genomic event that obliterates the clear demarcation between the large single-copy (LSC) and small single-copy (SSC) regions. Four independent major lineages within Papilionoideae (Fabaceae) are known to exhibit complete or partial IR loss and are collectively circumscribed as the Inverted Repeat-Lacking Clade (IRLC). This species-rich clade encompasses approximately 48 genera and more than 4,000 species, including *Astragalus* (Lee et al. 2021). The elimination of IR regions triggers a suite of profound evolutionary consequences. On the one hand, it releases the tight structural constraints on plastid genomes and promotes widespread genomic rearrangements, making IRLC plastomes the most structurally labile group documented in Fabaceae to date (Lee et al. 2021; Wu et al. 2021). On the other hand, it drastically accelerates genome-wide molecular evolutionary rates: substitution rates across all functional compartments in IRLC taxa are consistently and significantly elevated compared with those of closely related legume lineages that retain canonical IR copies (Schwarz et al. 2017). This unique and inherently unstable genomic architecture is hypothesized to have played a pivotal role in promoting genetic differentiation and high-altitude adaptive evolution in *A. baimaensis*.

### Phylogenetic position of *Astragalus baimaensis*

The phylogenomic analyses using two independent analytical pipelines indicate that *A. baimaensis* is closely related to *A. tongolensis* (Fig. 4) and *A. chilienshanensis* (Fig. 5). Multivariate morphological comparisons confirm that *A. baimaensis* differs consistently and significantly from all closely related species in pubescence, leaflet shape, flower color, and legume morphology (Table 2). These discrete diagnostic differences effectively rule out the hypothesis that the new taxon represents an extreme morphological variant of a known species and strongly corroborate its status as an evolutionarily distinct, previously undescribed lineage. Consistently, both ML and BI analyses performed in this study recovered fully concordant topologies that placed *A. baimaensis* within *Astragalus* sect. Cenantrum. Critically, *A. tongolensis*—a well-documented member of sect. Cenantrum—not only occurs sympatrically with *A. baimaensis* across the Baima Mountain range but also shares several key morphological traits with the new species, providing independent corroboration of their close phylogenetic affinity. Finally, all diagnostic sectional characters of sect. Cenantrum—including subsessile leaves, long-ascending pedunculate racemes, multiflorous inflorescences, campanulate calyces, yellow petals that darken upon desiccation, and papery legume valves—are fully exhibited by *A. baimaensis*, leaving no phenotypic contradiction to its sectional placement. Collectively, these lines of evidence strongly support the classification of *A. baimaensis* within sect. Cenantrum.

**Table 2.** Morphological comparative analysis of *Astragalus baimaensis* and its closely related congeners.

**Characters**	***A. baimaensis***	***A. tongolensis***	***A. chilienshanensis***	***A. acaulis***	***A. luculentus***
Plant height	plants 20–50 cm tall completely glabrous	plants 30–70(–120) cm tall, sparsely hairy	plant 20–30 cm tall	plant 15 cm tall	plant ca. 35 cm tall, completely glabrous
Stems	mostly several, erect, glabrous, angulate, 2–6 mm in diameter	several, up to 7 mm thick, glabrous	4–6 cm long, sparsely hairy	extremely short, usually not exceeding 3 cm, and glabrous	stout, glabrous, 3–4 mm in diam., ca. 20 cm
Leaves	3–9 cm	6–15 cm	4–8 cm	8–15 cm	25–30 cm
Leaflets	11–17, obovate, narrowly oblong, 10–22 × 4–8 mm, glabrous on both surfaces, base asymmetric, apex obtuse and mucronulate	7–15, narrowly elliptic to obovate, 10–60 × 4–25 mm, glabrous on both surfaces or some time abaxially loosely covered with nearly spreading white hairs 0.5–1 mm, apex obtuse	9–13, ovate to elliptic, 10–20 × 4–10 mm, glabrous or ciliate, apex rounded or slightly emarginate	15–25, narrowly ovate, measuring 6–8 × 2–3 mm, abaxially glabrous or only sparsely hairy along the margins and midvein, adaxially glabrous, apex is acute	23–31, ovate, 20–35 × 8–18 mm, apex subacute to rounded, often mi-nutely mucronulate
Petiole	0.5–1 cm long, glabrous	1–5 cm, like rachis glabrous or very sparsely hairy	2–4 cm	2–6 cm, like rachis glabrous or very sparsely hairy	8–11 cm
Stipules	triangular or ovoid, green to red purple, ca. 10-15 × 4–8 mm, glabrous	15–30 mm, sparsely ciliate	elliptic, 5–15 × 3–7 mm, white ciliate	white and membranous. upper stipules measure 10–12(–15) mm in length, the lower stipules are shorter, and all stipules are ciliate on the margins	20–30 mm, narrowly triangular, very long acuminate, distinctly longitudinally nerved, free from petiole
Inflorescence	racemes 8– to many-flowered, later strongly elongating	racemes rather densely 8- to many flowered, later strongly elongating	racemes 1–2 cm, ca. 10–flowered, elongating in fruit	racemes subsessile, 2– or 3–flowered	racemes distributed along whole stem
Peduncle	5–15 cm, red purple, sulcate, glabrous	6–25 cm, glabrous or sparsely white hairy, toward raceme also black hairy	15–20 cm, 2–3 times longer than the leaves	2–3 mm, glabrous	8–9 cm, loosely 5–9-flowered
Calyx	campanulate, ca. 7 mm long, glabrous, green to red purple and turn red purple at fruiting; teeth subulate, ca. 3 mm long, glabrous	turbinate-tubular, 6–9 mm, glabrous or sparsely hairy outside, on inner side in upper 1/2 of tube densely appressed black hairy, rarely also in upper 1/2 of outer side rather densely black hairy; teeth triangular, 1.5–2(–3) mm	campanulate, ca. 5 mm, glabrous; teeth narrowly triangular, ca. 2 mm, black hairy on inner side	tubular, 11–15 mm, tube glabrous or rarely sparsely hairy; teeth 3–4 mm, sometimes ciliate at margin and apex	12–13 mm; teeth unequal, 2–4 mm
Bracts	oblong or ovoid, green to red purple, 8–15 mm long, glabrous	whitish or greenish, linear, 5–10 mm, black ciliate, soon falling	linear-acute to narrowly ovate, 4–6 mm, sparsely white hairy	membranous, linear, acute, 10–15 mm, glabrous or ciliate	10–15 mm
Petals	green-yellow to yellow	yellow, all of equal length	yellow, all of nearly same length	pale sulfur-yellow	yellow
Standard	ca. 15 × 8 mm, obovate, without distinct claw, apex emarginate	oblong to slightly obovate, 18–24 × 6–8 mm, somewhat constricted in middle	widely obovate, 10–12 × ca. 9 mm	widely obovate, 20–25 × 11–13(–16) mm, apex rounded to slightly emarginate	ca. 25 mm
Wings	ca. 14 mm long, claw ca. 8 mm long, auricle ca. 1 mm long, blade oblong, ca. 6 × 2.5 mm, apex rounded	18 × 2.5 mm, claw ca. 13 mm	wings as long as standard	19–24 mm	–
Keel	ca. 14 mm long, claw ca. 8 mm long	17 × 3 mm, claw ca. 13 mm	keel as long as standard	19–22 mm	ca. 15 mm
Ovary	ovary with a stipe ca. 1.5 mm, glabrous; style and stigma glabrous	ovary is narrowly ovoid, stipitate, densely covered with black and white pubescence; style and stigma glabrous	ovary is narrowly ovate, densely covered with white and black pubescence, and stipitate	ovary linear, glabrous, stipitate	–
Legumes	legumes with a stipe ca. 10 mm, inflated, obliquely ellipsoid, 20–30 × 6–10 mm, with a beak 2 mm; valves membranous, glabrous, turn red purple at fruiting	legumes with a stipe 8–10 mm, narrowly ovoid, 20–25 mm × 5–6 mm, keeled ventrally, flattened dorsally, apex long acuminate; valves papery, very densely black hairy	legumes with a stipe ca. 3 mm, pendulous, narrowly ellipsoid, ca. 20 mm, attenuate at both ends; valves sparsely black hairy	legumes subsessile, erect, slightly obliquely oblong, 25–50 mm, 10–15 mm high and 3–4 mm wide, narrowly rounded ventrally and dorsally, without beak, nearly completely 2–locular glabrous	Legumes subsessile, oblong, straight, 18–25 mm, 5–8 mm high and ca. 9 mm wide, rounded with prominent, thickened nerve ventrally, deeply and widely grooved dorsally, with a narrowly triangular beak 1–2 mm, incompletely 2–locular; valves leath-ery
Distribution	Yunnan	Gansu, Qinghai, Sichuan, Xizang, Yunnan	Qinghai, Xizang	Sichuan, Xizang, Yunnan [Bhutan, India (Sikkim)]	Xinjiang

Note: All characters were documented based on observations of the specimens and described in accordance with the information provided in Flora of China (Xu and Podlech 2010) and Flora Reipublicae Popularis Sinicae (Ho and Fu 1993).

Phylogenetic analyses based on the complete plastome resolved the newly described species *Astragalus baimaensis* as sister to *A. tongolensis*, whereas the combined ITS + *rbcL* + *matK* multilocus phylogeny recovered *A. chilienshanensis* as its closest extant relative. Two non-mutually exclusive mechanisms may explain this topological discordance. First, recurrent chloroplast capture events have been well documented in plant lineages endemic to the Hengduan Mountains, as recently reported in *Taxus* (Qin et al. 2023). It is therefore parsimonious to infer that the plastome of *A. baimaensis* was acquired through historical introgressive hybridization with *A. tongolensis* or its direct ancestor, whereas the nuclear ribosomal ITS region retains a closer phylogenetic affinity to *A. chilienshanensis*. Second, the full plastome dataset (ca. 120 kb) contains approximately 40 times more phylogenetically informative characters than the three-locus combined alignment (ca. 3 kb), which inherently provides considerably higher phylogenetic resolution. Critically, the plastome is strictly maternally inherited in most Leguminosae and thus captures only a single evolutionary lineage, whereas the concatenated ITS + *rbcL* + *matK* dataset integrates both biparentally inherited nuclear (ITS) and maternally inherited plastid (*rbcL* and *matK*) markers that carry distinct evolutionary signals. Such widespread cytonuclear phylogenetic discordance is not anomalous and has been extensively documented across Fabaceae, with conflicting topologies frequently recovered at multiple taxonomic nodes when marker sets with different inheritance modes are employed (Zhang et al. 2026).

Exclusive reliance on molecular data alone is insufficient to rigorously resolve fine-scale species boundaries within the species-rich, morphologically heterogeneous genus *Astragalus*. The phylogenomic analyses implemented in the present study consistently failed to recover reciprocal monophyly for several well-established sections circumscribed in Flora of China, particularly sect. Cenantrum. This nonmonophyletic pattern is fully concordant with previous, widely validated taxonomic findings across the genus (Bagheri et al. 2023), confirming that the current morphology-based infrageneric classification system does not accurately reflect the full evolutionary history of *Astragalus*. Traditionally, infrageneric sectional delimitation of *Astragalus* has relied on a restricted set of macromorphological diagnostic characters, among which legume and foliar traits are the most widely applied. Under persistent strong environmental selection pressure, these functional morphological characters exhibit exceptionally high levels of homoplasy (Naderi Safar et al. 2014), leading to widespread phenotypic convergence among distantly related species that do not share a most recent common ancestor. As a direct consequence, classification frameworks founded solely on morphological evidence systematically underestimate the true evolutionary complexity of the genus. By synthesizing phylogenomic, morphological, and ecological lines of evidence, the total-evidence integrative taxonomic paradigm not only substantially increases the robustness of species delimitation but also establishes a more comprehensive analytical framework for disentangling the full spectrum of evolutionary relationships and cryptic species diversity in *Astragalus* (Borges et al. 2013). Such a multi-evidence integrative approach provides a rigorous foundation for advancing systematic studies of the genus, offering both depth and breadth for elucidating its highly complex taxonomic relationships and evolutionary history.

### Ovary pubescence as a reliable trait for species identification in *Astragalus*

Within *Astragalus*, a genus characterized by extraordinary morphological diversification, trichome traits—including indumentum type, density, and topographic distribution—are widely recognized as taxonomically decisive diagnostic markers (Su et al. 2021). In this study, all aerial organs of *A. baimaensis*, including the stems, leaves, leaflets, bracts, calyces, and even petals, were entirely glabrous (Fig. 7). Most critically, the ovaries of *A. baimaensis* were completely glabrous, a trait that showed no intraspecific variation among all 50 mature individuals sampled throughout its known distribution on Baima Mountain (Fig. 7). When directly compared with its closely related sympatric congeners *A. tongolensis* and *A. chilienshanensis*, this glabrous ovary trait immediately distinguishes the new species: both *A. tongolensis* and *A. chilienshanensis* bear dense black or white trichomes on their ovaries, providing unambiguous morphological support for the evolutionary independence of the three taxa (Table 2). A parallel taxonomic precedent can be found in the Flora of China treatment, which formerly merged *A. membranaceus* (Fisch.) Bunge into *A. mongholicus* Bunge (Xu and Podlech 2010). However, a consistent morphological discontinuity persists between the two entities: *A. membranaceus* has uniformly black-pubescent ovaries, whereas *A. mongholicus* has glabrous or sparsely strigose ovaries with rare, very short, appressed black or white hairs (Ho and Fu 1993). Moreover, results from two independently performed phylogenetic inference methods consistently support the status of *A. membranaceus* and *A. mongholicus* as well-separated, distinct species in the phylogenetic trees (Figs 4, 5, 6). Crucially, the ovary trichome phenotype exhibits perfect developmental correlation with legume indumentum: species with pubescent ovaries invariably produce pubescent legumes. This fruit pubescence trait has long served as a section-level diagnostic character in *Astragalus* infrageneric classification. Consistent with the taxonomic treatment in Flora of China, this highly stable indumentum trait alone is sufficient to clearly distinguish sects Nuculiella and Myobroma, as well as sects Lithoon and Lithophili, from other phylogenetically distant clades (Xu and Podlech 2010). The combined morphological observations, phylogenomic results, and established taxonomic precedent indicate that the presence or absence of ovary pubescence represents an exceptionally stable, low-homoplasy diagnostic character for species delimitation in *Astragalus*. This trait effectively reduces the taxonomic ambiguity that commonly arises from overreliance on phenotypically plastic macromorphological features such as leaf outline and flower color.

## Conclusion

Based on the targeted field surveys conducted to date, *Astragalus baimaensis* exhibits an extremely narrow endemic distribution, restricted to alpine scrub and subnival meadow habitats at elevations of 4,200–4,400 m exclusively on Baima Mountain (Figs 1, 2). This highly geographically restricted distribution pattern underscores its specialized ecological niche and intrinsic population vulnerability. Notably, this narrow-range endemic profile is fully consistent with the well-documented status of the Hengduan Mountains as a biodiversity hotspot, widely recognized as one of the primary centers of recent species radiation and diversification within *Astragalus* (Zhang et al. 2021). The region’s intricately dissected topography, steep elevational gradients, and exceptionally heterogeneous microclimates generate an ideal combination of geographic isolation and ecological filtering required for allopatric speciation (Sun et al. 2017). Through long-term local adaptation to the harsh high-altitude alpine environment, *A. baimaensis* has presumably evolved a suite of distinct adaptive traits. These traits include a dwarf growth habit and putatively unique physiological mechanisms that collectively enable the species to tolerate intense solar radiation, low temperatures, and an extremely short growing season at high elevations (Tian et al. 2023). Furthermore, the unique morphology of its pods—characterized by a long stipe and pendulous orientation—likely represents an adaptive seed-dispersal strategy suited to the strong winds prevalent in high-altitude environments (Wang et al. 2024; Sun et al. 2025). Overall, *A. baimaensis* not only highlights the pivotal role of regional environmental factors in driving species formation but also, owing to its restricted distribution and highly specialized ecological niche, represents an ideal model for investigating the adaptive mechanisms of *Astragalus* species in alpine environments.

## Supplementary Material

XML Treatment for Astragalus baimaensis

## Additional information

### Artificial Intelligence (AI) use

The authors accept full responsibility for the content of the manuscript, including the disclosure of any use of AI.

No AI tools were used in the preparation of this manuscript.

### Author ORCIDs

Q.-Y. Chen https://orcid.org/0009-0008-9328-9187

H.-T. Ma https://orcid.org/0009-0009-5057-2780

Y.-Z. Zhang https://orcid.org/0009-0002-5992-1739

### Data availability

All of the data that support the findings of this study are available in the main text or Supplementary Information.

## Supplementary materials

Supplementary material 1 Tables and figures Data type docx Explanation note **table SS1**. Sample information of gene fragments used for phylogenetic inference. **table SS2**. Information on the chloroplast genomes used for phylogenetic inference. **fig. S1**. Holotype of *Astragalus baimaensis*. **fig. S2**. Isotype. **fig. S3**. Paratype. **fig. S4**. Paratype. https://binary.pensoft.net/file/1731362This dataset is made available under the Open Database License (http://opendatacommons.org/licenses/odbl/1.0/). The Open Database License (ODbL) is a license agreement intended to allow users to freely share, modify, and use this Dataset while maintaining this same freedom for others, provided that the original source and author(s) are credited. Qi-Yin Chen, Hai-Tao Ma, Jie-Hua Rao, Hai-Feng Li, Bei Jiang, Zhuo Zhou, Yong-Zeng Zhang
