# Supplementary material for: Astragalus baimaensis (Fabaceae), a new species from Yunnan, China
Source: PhytoKeys. 2026 Jul 30;278:55–74. doi: 10.3897/phytokeys.278.191151 (PMC13448472; doi:10.3897/phytokeys.278.191151)
Supplement: Supplementary file 1 [file phytokeys-278-055_article-191151__-s001.docx]

**Table S1.** Sample information of gene fragments used for phylogenetic inference in this study. Sequences newly generated in this study are highlighted in bold font. (- indicates that the sample gene fragment information is obtained from its complete chloroplast genome data.)

| **Species** | **Family** | **Genus** | **ITS** | ***rbcL*** | ***matK*** |
| --- | --- | --- | --- | --- | --- |
| ***Astragalus baimaensis*** **B. Jiang & Y. Z. Zhang** | **Fabaceae** | ***Astragalus*** | **PX747761.1** | - | - |
| ***Astragalus tongolensis* Ulbr.** | **Fabaceae** | ***Astragalus*** | **PX747760.1** | - | - |
| *Astragalus membranaceus* (Fisch.) Bunge | Fabaceae | *Astragalus* | JX017331.1 | MN729557.1 | MN746763.1 |
| *Astragalus mongholicus* (Bunge) P. K. Hsiao | Fabaceae | *Astragalus* | MN224267.1 | MN224286.1 | MT918211.1 |
| *Astragalus ernestii* H. F. Comber | Fabaceae | *Astragalus* | MN224276.1 | MN224297.1 | MH293199.1 |
| *Astragalus monadelphus* Bunge ex Maxim. | Fabaceae | *Astragalus* | MH808433.1 | MN192624.1 | MN311770.1 |
| *Astragalus chilienshanensis* Y. C. Ho | Fabaceae | *Astragalus* | MW432232.1 | MW409221.1 | MW417528.1 |
| *Astragalus lithophilus* Kar. & Kir. | Fabaceae | *Astragalus* | MT923542.1 | MT931368.1 | MT918213.1 |
| *Astragalus melilotoides* Pall. | Fabaceae | *Astragalus* | MH808431.1 | MN204886.1 | MN311769.1 |
| *Astragalus skythropos* Bunge | Fabaceae | *Astragalus* | MH808437.1 | MN192625.1 | MN311772.1 |
| *Astragalus yunnanensis* Franch. | Fabaceae | *Astragalus* | MH293132.1 | MH293273.1 | MH293205.1 |
| *Astragalus chinensis* L. f. | Fabaceae | *Astragalus* | AF121681.1 | GQ436350.1 | HM142263.1 |
| *Astragalus tumbatsica* Marq. et Airy-Shaw | Fabaceae | *Astragalus* | MF044313.1 | MF044425.1 | MF044370.1 |
| *Astragalus alpinus* L. | Fabaceae | *Astragalus* | KX954891.1 | MF158749.1 | KC474146.1 |
| *Astragalus kuschakevitschii* B. Fedtsch. ex O. Fedtsch. | Fabaceae | *Astragalus* | MT923538.1 | MT931364.1 | MT946437.1 |
| *Astragalus retusifoliatus* Y. C. Ho | Fabaceae | *Astragalus* | MF044265.1 | MF044378.1 | MF044322.1 |
| *Astragalus sinicus* L. | Fabaceae | *Astragalus* | MH808435.1 | MN204888.1 | MN433358.1 |
| *Astragalus acaulis* Baker | Fabaceae | *Astragalus* | MH293125.1 | MH293265.1 | MH293198.1 |
| *Astragalus flexus* Fisch. | Fabaceae | *Astragalus* | PQ590553.1 | OR700268.1 | MT918242.1 |
| *Astragalus forrestii* N. D. Simpson | Fabaceae | *Astragalus* | MF044310.1 | MF044422.1 | MF044367.1 |
| *Astragalus polycladus* Bureau & Franch. | Fabaceae | *Astragalus* | MF044276.1 | MF044389.1 | MF044333.1 |
| *Astragalus Pullus* N. D. Simpson | Fabaceae | *Astragalus* | MT914287.1 | MF044413.1 | MT928692.1 |
| *Astragalus dasyglottis* Fisch. ex DC. | Fabaceae | *Astragalus* | MT923530.1 | MT931356.1 | MT918210.1 |
| *Astragalus tibetanus* Benth. ex Bunge | Fabaceae | *Astragalus* | OQ106946.1 | MT885865.1 | MW110816.1 |
| *Astragalus strictus* Graham | Fabaceae | *Astragalus* | PX433244.1 | MF786767.1 | MF786992.1 |
| *Astragalus arpilobus* Kar. & Kir. | Fabaceae | *Astragalus* | MT923527.1 | MT931353.1 | MT918236.1 |
| *Astragalus tribuloides* Delile | Fabaceae | *Astragalus* | KX955042.1 | KY951682.1 | KX955200.1 |
| *Astragalus alopecurus* Pall. | Fabaceae | *Astragalus* | MF543756.1 | MF572163.1 | MF543487.1 |
| *Astragalus vulpinus* Willd. | Fabaceae | *Astragalus* | PQ167957.1 | OR738177.1 | MT931381.1 |
| *Astragalus dilutus* Bunge | Fabaceae | *Astragalus* | MT923532.1 | MT931358.1 | MT918212.1 |
| *Astragalus cognatus* C. A. Mey. | Fabaceae | *Astragalus* | MZ366382.1 | MZ361385.1 | MT918215.1 |
| *Astragalus gebleri* Fisch. ex Bong. | Fabaceae | *Astragalus* | MT923537.1 | MT931363.1 | MT918224.1 |
| *Astragalus bhotanensis* Baker | Fabaceae | *Astragalus* | MH808429.1 | MN185154.1 | MN433356.1 |
| *Astragalus adsurgens* Pall. | Fabaceae | *Astragalus* | MN871858.1 | MH657731.1 | MH659288.1 |
| *Astragalus lasiophyllus* Ledeb. | Fabaceae | *Astragalus* | MT923539.1 | MT931365.1 | MT918221.1 |
| *Astragalus scaberrimus* Bunge | Fabaceae | *Astragalus* | MT923549.1 | MT931375.1 | MT918219.1 |
| *Astragalus scabrisetus* Bong. | Fabaceae | *Astragalus* | MT923550.1 | MT931376.1 | MT918216.1 |
| *Astragalus arbuscula* Pall. | Fabaceae | *Astragalus* | MT923521.1 | MT931347.1 | MT918223.1 |
| *Caragana arborescens* Lam. | Fabaceae | *Caragana* | PQ167875.1 | PQ337490.1 | PQ348634.1 |
| *Caragana jubata* (Pall.) Poir. | Fabaceae | *Caragana* | PQ763931.1 | MW382763.1 | MW382586.1 |

**Table S2.** Information on the chloroplast genomes used for phylogenetic inference in this study. Sequences newly generated in this study are highlighted in bold font.

| **Species** | **Family** | **Genus** | **GenBank number** |
| --- | --- | --- | --- |
| ***Astragalus baimaensis* B. Jiang & Y. Z. Zhang** | **Fabaceae** | ***Astragalus*** | **PX754581.1** |
| ***Astragalus tongolensis* Ulbr.** | **Fabaceae** | ***Astragalus*** | **PX754582.1** |
| *Astragalus floridus* Benth. ex Bunge | Fabaceae | *Astragalus* | NC_083391.1 |
| *Astragalus stipulatus* D. Don ex N. D. Simpson | Fabaceae | *Astragalus* | OR491698.1 |
| *Astragalus membranaceus* (Fisch.) Bunge | Fabaceae | *Astragalus* | OR528897.1 |
| *Astragalus leucocephalus* Graham | Fabaceae | *Astragalus* | NC_085734.1 |
| *Astragalus acaulis* Baker | Fabaceae | *Astragalus* | PV802397.1 |
| *Astragalus psilosepalus* Podlech & L. R. Xu | Fabaceae | *Astragalus* | NC_083910.1 |
| *Astragalus galactites* Pall. | Fabaceae | *Astragalus* | NC_058825.1 |
| *Astragalus odoratus* Lam. | Fabaceae | *Astragalus* | LC764837.1 |
| *Astragalus mesoleios* Boiss. & Hohen. | Fabaceae | *Astragalus* | LC764836.1 |
| *Astragalus macropelmatus* Bunge | Fabaceae | *Astragalus* | LC764835.1 |
| *Astragalus iranicus* Bunge | Fabaceae | *Astragalus* | LC764834.1 |
| *Astragalus nakaianus* Y.N.Lee | Fabaceae | *Astragalus* | NC_028171.1 |
| *Astragalus oxyglottis* Steven | Fabaceae | *Astragalus* | NC_077543.1 |
| *Astragalus pallasii* Spreng. | Fabaceae | *Astragalus* | NC_077542.1 |
| *Astragalus agrestis* G. Don | Fabaceae | *Astragalus* | NC_077551.1 |
| *Astragalus arbuscula* Pall. | Fabaceae | *Astragalus* | NC_077550.1 |
| *Astragalus cognatus* C. A. Mey. | Fabaceae | *Astragalus* | NC_077548.1 |
| *Astragalus canadensis* var. brevidens (Gand.) Barneby | Fabaceae | *Astragalus* | NC_060799.1 |
| *Astragalus gummifer* Labill. | Fabaceae | *Astragalus* | NC_047251.1 |
| *Astragalus jiuquanensis* S. B. Ho | Fabaceae | *Astragalus* | NC_077545.1 |
| *Astragalus arpilobus* Kar. & Kir. | Fabaceae | *Astragalus* | NC_077549.1 |
| *Astragalus dilutus* Bunge | Fabaceae | *Astragalus* | ON550407.1 |
| *Astragalus flexus* Fisch. | Fabaceae | *Astragalus* | ON550405.1 |
| *Astragalus khasianus* Bunge | Fabaceae | *Astragalus* | PV798172.1 |
| *Astragalus laxmannii* Jacq. | Fabaceae | *Astragalus* | NC_085710.1 |
| *Astragalus lithophilus* Kar. & Kir. | Fabaceae | *Astragalus* | NC_077544.1 |
| *Astragalus melilotoides* Pall. | Fabaceae | *Astragalus* | NC_072247.1 |
| *Astragalus mongholicus* Bunge | Fabaceae | *Astragalus* | OR712437.1 |
| *Astragalus muliensis* Hand.-Mazz. | Fabaceae | *Astragalus* | OR491696.1 |
| *Astragalus polycladus* var. nigrescens (Franch.) E. Peter | Fabaceae | *Astragalus* | PV910879.1 |
| *Astragalus polycladus* Bureau & Franch. | Fabaceae | *Astragalus* | PV910878.1 |
| *Astragalus purpurinus* (Y. C. Ho) Podlech & L. R. Xu | Fabaceae | *Astragalus* | PP937056.1 |
| *Astragalus scabrisetus* Bong. | Fabaceae | *Astragalus* | NC_077541.1 |
| *Astragalus sieversianus* Pall. | Fabaceae | *Astragalus* | NC_077540.1 |
| *Astragalus sinicus* L. | Fabaceae | *Astragalus* | OM287552.1 |
| *Astragalus strictus* Graham | Fabaceae | *Astragalus* | MT120746.1 |
| *Astragalus tenuis* Turcz. | Fabaceae | *Astragalus* | OP723862.1 |
| *Astragalus tumbatsica* Marq. et Airy-Shaw | Fabaceae | *Astragalus* | NC_083912.1 |
| *Astragalus vulpinus* Willd. | Fabaceae | *Astragalus* | NC_077539.1 |
| *Astragalus yunnanensis* Franch. | Fabaceae | *Astragalus* | PV156652.1 |
| *Astragalus yunnanensis* subsp. *incanus* (E. Peter) Podlech & L. R. Xu | Fabaceae | *Astragalus* | PV156653.1 |
| *Caragana arborescens* Lam. | Fabaceae | *Caragana* | MT211962.1 |
| *Caragana jubata* (Pall.) Poir. | Fabaceae | *Caragana* | MT211963.1 |

**Figure S1.** Holotype of *Astragalus baimaensis* B. Jiang & Y. Z. Zhang, sp. nov.

**Figure S2.** Isotype of *Astragalus baimaensis* B. Jiang & Y. Z. Zhang, sp. nov.

**Figure S3.** Paratype of *Astragalus baimaensis* B. Jiang & Y. Z. Zhang, sp. nov.

**Figure S4.** Paratype of *Astragalus baimaensis* B. Jiang & Y. Z. Zhang, sp. nov.
